# Choose and stay on one out of two paths: distinction between clinical versus research genetic testing to identify cancer predisposition syndromes among patients with cancer

**DOI:** 10.1007/s10689-021-00228-2

**Published:** 2021-02-12

**Authors:** Tim Ripperger, D Gareth Evans, David Malkin, Christian P. Kratz

**Affiliations:** 1 Department of Human Genetics, Hannover Medical School, Hannover, Germany; 2 Faculty of Biology, Medicine and Health, Division of Evolution and Genomic Sciences, School of Biological Sciences, University of Manchester, Manchester Academic Health Science Centre, Manchester, M13 9PL UK; 3 Division of Hematology/Oncology, Department of Pediatrics, The Hospital for Sick Children, University of Toronto, Toronto, ON Canada; 4 Department of Pediatric Hematology and Oncology, Hannover Medical School, Hannover, Germany

Specific constitutional (epi)genetic alterations are known to cause cancer predisposition syndromes (CPS) and represent confirmed cancer risk factors [1–4]. The cancer risks and spectra as well as benign phenotypes associated with various CPS are diverse and characterized by interfamilial and intrafamilial variability [1–3]. Hence, the identification of patients with a CPS is not trivial. Furthermore, the diagnosis of a CPS in a patient with cancer may have broad clinical implications with regard to counseling, prevention, surveillance, therapy, psychosocial support, and identification of relatives at risk [1–4].

The methods employed to search for causative (epi)genetic alterations in individuals with a suspected CPS have dramatically improved during the last decade. While this technological progress is associated with multiple benefits, there are also potential disadvantages. Diagnostic application of large non-specific CPS gene panels or agnostic whole exome or even genome sequencing are linked with (higher) probabilities of (a) detecting variants of uncertain significance (VUS), (b) variants in CPS candidate genes or accepted CPS genes with non-proven causality regarding the cancer of interest, and (c) secondary findings not linked to the clinical question that led to the genetic test. Increasingly, the diagnosis of a specific CPS is made purely based on the detection of a given (epi)genetic variant in the absence of typical clinical features associated with the specific CPS. Historically, the diagnosis of a CPS was made based on the presence of typical clinical features and confirmed through the detection of a causative (epi)genetic alteration. Surveillance strategies and other measures have been developed in the past for patients meeting the clinical criteria. However, it is not known whether the broad clinical implications associated with the diagnosis of a CPS are justifiable in the absence of respective clinical features.

In order to diagnose patients with cancer who have an underlying CPS, various strategies are being employed. These strategies differ with regard to two questions: (1) who is being offered counseling and testing? and (2) which method is employed for genetic analysis? In order to answer both questions, we recommend to distinguish between a *clinical* and a *research path*. Insufficient separation can lead to unsolved issues and unforeseen problems in clinical care. In addition, handling of incidental and secondary findings have to be defined and communicated. Secondary findings are commonly defined as known clearly pathogenic gene variants (ACMG class 4/5) in the available genetic dataset which are actively and specifically searched for but for which no association with the diagnostic study objective is given. Incidental findings are also not *a priori* associated with the focus of the genetic investigation but have been identified as a byproduct when filtering the available dataset for possibly study-related gene variants [5].

## Clinical testing strategy

When following this path, genetic testing is offered only to those patients meeting specific clinical criteria for a given CPS followed by focused genetic testing [6]. The use of clinical screening tools such as questionnaires [1, 7] or mobile apps [8] may help to identify patients in whom genetic testing may be indicated. Following this preselection process, it is carefully evaluated whether genetic counseling and testing are indicated and which (epi)genetic analyses should be considered. Following counseling and diagnostic testing, only ACMG class 3 (variant of unknown significance (VUS)), 4 (likely pathogenic) and 5 (pathogenic) variants affecting genes of interest (i.e., genes with a known causal association with the clinical situation being examined) are communicated. If, due to technological reasons (e.g., use of gene panels vs. whole exome sequencing platforms), additional genes are investigated, one feasible (and our preferred) option is to focus on the gene(s) of interest only and to purposefully ignore (i.e., not search for and not report) findings (i.e., secondary/incidental findings) not relevant to the disease and signs/symptoms being evaluated, because the significance and potential benefit of such findings to patient management may be questionable in the absence of clinical features. To diagnostically focus on gene(s) of interest in the era of routinely applied large gene panels or whole exome/genome sequencing, we prefer strict filtering and thus, focus on variants in previously defined genes of interest with a known causative association. Ideally, the clinical testing strategy continuously evolves and improves as new research-generated findings challenge existing criteria and are translated to the clinical ‘pipeline’.

## Research testing strategy

This strategy aims at knowledge expansion; as such, it may benefit from a less stringent patient selection algorithm followed by more agnostic testing methods to increase the probability of identifying novel CPS genes as well as CPS patients who did not meet the currently known or accepted testing criteria [9]. On the other hand, this approach may be associated with discovery of findings that are not necessarily beneficial to the tested individual. Examples include: (a) a higher likelihood of detecting VUSs, (b) identification of (likely) pathogenic variants in CPS genes with an unproven role in the pathogenesis of the cancer type that prompted the genetic analysis, and (c) incidental and/or secondary findings in the absence of signs or symptoms of that specific condition. In all three situations, tested individuals and clinicians may potentially be confronted with unforeseen dilemmas, despite the unproven benefit associated with this genetic information. This becomes even more problematic in minors, for whom consent for genetic testing was provided by their parents or guardians, overriding their autonomy. These issues have to be clearly addressed during informed consent clearly defining which information can be communicated when and how.

## Testing outside clinical care

Of course, the above deliberately does not allude to the increasing number of patients and potentially parents of children with cancer who choose to access direct-to- consumer testing, which often includes testing of many genes beyond those that are clinically indicated.

## Stay on one path

**Fig. 1:**
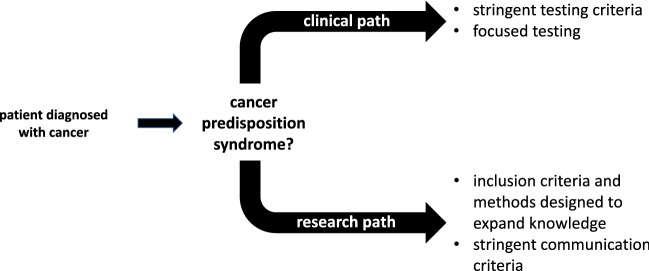
Clinical and research paths to investigate the presence of cancer predisposition syndromes among patients with cancer

The ongoing constant improvement of sequencing technologies has greatly expanded our knowledge in the field of cancer predisposition. Prior to genetic testing of an individual, we recommend choosing between a *clinical path* that is based on known accepted testing criteria and a *research path* that aims at making novel discoveries (Fig. 1). Both paths are currently often intermixed and tested individuals are being confronted with genetic information that may cause more harm than benefit. Therefore, we recommend that testing outside established criteria be offered only within defined research projects that incorporate careful strategies whereby only carefully selected and clinically meaningful variants that are identified can be validated in a clinically-certified laboratory and the results communicated and translated into clinical practice. Ideally, participants of such research are followed prospectively to study the clinical significance of any novel findings.
